# Cyclopædia of the Practice of Medicine

**Published:** 1877-12

**Authors:** 


### BIBLIOGRAPHICAL.

Cyclopædia of the Practice of Medicine. Edited by Dr. H. Von Ziemssen, Professor of Clinical Medicine, Munich, Bavaria.

The twelfth volume of this immense work, edited by Albert H. Buck, M.D., from the publishers, Wm. Wood & Co., New York, was received at this office two months since. In the November number of our Journal its reception was acknowledged, and mention was made of the authors furnishing the matter it contains. We did no more from press of time, nor did any thing further strike us as important to say of a work so lavishly praised already by journalists and the profession generally. Besides, German medical literature while it leads us to great perfection in some departments of the healing art, is, as this work clearly demonstrates, sadly deficient in others. Exhaustive discussion on pathology and etiology, particularly the former, is had in the volume before us, connected with the diseases of which it treats. The national tendencies in Paris, London, Edinburgh and Dublin are to the study of medical and surgical therapeutics, while Berlin, Munich, Heidelberg and Leipsic are devoted to pathological investigations. The scalpel and microscope are to the Germans what the ecraseur and alkaloids are to the French and English. It is playfully said that the German would like to see his patient die, that he might examine the diseased structures.

Dr. Zeimssen's authors certainly exhibit the national proclivity in their very thorough pathological research. For example, in the chapter on inflammations of the dura-mater, fifty pages are devoted to pathology, &c., and only two to the treatment.

To Americans, whose education in pathology is, too often, sadly deficient, this German work is, doubtless, acceptable. It should be peculiarly so to such as have been all their lives in hot haste to drench their patients with every obtainable drug, without definite knowledge of the pathological state, or the physiological actions of remedies.

For a complete library on the practice of medicine, however, we prefer more of remedies and their action, than is found in Ziemssen's Encyclopædia.

A notable instance of the opposite extremes, to which German and American writers are subject, is found in the above work and that of the following:

